# Evidence of Circulation of Several HAV Genetic Variants and Emergence of Potential Antigenic Variants in an Endemo-Epidemic Country before Vaccine Introduction

**DOI:** 10.3390/v13061056

**Published:** 2021-06-03

**Authors:** Kaouther Ayouni, Anissa Chouikha, Oussema Khamessi, Henda Touzi, Walid Hammemi, Henda Triki

**Affiliations:** 1 Laboratory of Clinical Virology, WHO Reference Laboratory for Poliomyelitis and Measles in the Eastern Mediterranean Region, Pasteur Institute of Tunis, University Tunis El Manar (UTM), Tunis 1002, Tunisia; touzihenda@yahoo.fr (H.T.); Hammemi.walid12@gmail.com (W.H.); henda.triki@pasteur.tn (H.T.); 2 Faculty of Sciences of Tunis, University of Tunis El Manar, Campus Universitaire, El Manar, Tunis 2092, Tunisia; 3 Institut Pasteur de Tunis, Université de Tunis El Manar, LR11IPT08 Venins et Biomolecules Therapeutiques, Tunis 1002, Tunisia; oussama.khamassi@pasteur.tn; 4 Faculty of Medicine of Tunis, University of Tunis El Manar, 15 Rue Djebel Lakhdhar, La Rabta, Tunis 1007, Tunisia

**Keywords:** hepatitis A virus, molecular characterization, antigenic variant, genotyping, public health

## Abstract

Similar to several other countries in the world, the epidemiology of hepatitis A virus changed from high to intermediate endemicity level in Tunisia, which led to the occurrence of outbreaks. This study aimed to determine the genetic and antigenic variability of HAV strains circulating in Tunisia during the last few years. Genotyping using complete VP1 gene and VP1-2A junction confirmed the predominance of genotype IA, with co-circulation of several genetic and antigenic variants. Phylogenetic analysis including Tunisian and strains from other regions of the world showed the presence of at least two IA-variants within IA subgenotype. Amino-acid analysis showed several mutations in or close to epitope regions in the VP1-region. This study provides a baseline on the genetic and antigenic variability of HAV circulating strains before the introduction of vaccination into the national immunization schedule.

## 1. Introduction

Hepatitis A infection remains a major public health problem despite the presence of effective vaccines since 1992. The infection is distributed worldwide but occurs more frequently in less developed regions with poor hygienic and sanitary conditions, causing substantial morbidity and mortality rates [1,2]. WHO estimates that hepatitis A caused approximately 7134 deaths in 2016, which accounts for 0.5% of the mortality worldwide due to viral hepatitis [3].

Hepatitis A infection is caused by hepatitis A virus (HAV), an RNA virus classified within the *Picornaviridae* family and Hepatovirus genus. HAV exists in a dual phenotype, naked and quasi-enveloped virions [4]. The quasi-enveloped virions represent the immature particles that are found in the blood, whereas the naked virions in feces are those released form their quasi-envelope by the action of bile salts in the passage from the bile ducts to the gut [4,5,6]. The naked virions are mature and contain the fully processed VP1 protein [4,5,6]. HAV genome is a positive single stranded RNA of approximately 7.5 Kb which comprises a single open reading frame (ORF) and two untranslated regions on its 3′and 5′ ends. Genotyping analysis of different HAV genomic regions revealed the existence of 6 different genotypes [7,8,9,10]. Genotypes I to III, sub-divided into sub-genotype IA, IB, IC, IIA, IIB, IIIA and IIIB, infect humans, while genotypes IV to VI are of simian origin [11,12]. 

The severity of the disease is age dependent. Unlike young children, teenagers and adults usually develop symptomatic clinical forms, sometimes with high severity [13,14]. HAV infection is more common in developing countries, most children get infected before the age of 10 years and older children and adults are generally immune. In these countries, symptomatic disease rates are low and outbreaks are uncommon. In countries with higher economic level, the HAV infection occurs in older ages and symptomatic cases as well as outbreaks are more frequent. During the past years, an epidemiological change of hepatitis A pattern was observed in many parts of the world [15,16,17,18,19,20,21,22,23,24]. Globally, an increase of symptomatic cases was observed [25,26]. Several outbreaks have been triggered in countries whose epidemiological status has changed from high to medium or low endemicity with more severe outcomes [25,27]. Thus, the implementation of programs for HAV surveillance and the introduction of HAV vaccine stand out as the best measure to limit and prevent the spread of the virus, especially in countries with intermediate or low endemicity levels.

In Tunisia, a transition from high to intermediate endemicity level was observed [28,29,30]. Improvement of socioeconomic and hygienic conditions had contributed to the decreasing frequency of the infection among young children, especially among primary school attendants, and led to serious outbreaks and significant disease burden. The national health authorities have committed, in line with the World Health Organization’s (WHO) resolution, to eliminate hepatitis viruses by 2030 as a public health threat. In this context, novel measures to limit the transmission of the disease were undertaken, by implementing an HAV surveillance program and introducing HAV vaccine. In October 2018, HAV vaccination was introduced for 6-year-old children at school entry. Since September 2020, the national HAV vaccination schedule has combined a single dose at 12 months with a catch-up vaccination for non-vaccinated children aged 6 years.

The knowledge of the molecular epidemiology of viral strains circulating is a key component to evaluate the vaccine efficiency, to follow progression of the national program and to detect a possible emergence of vaccine-escape variants. Only few studies have investigated the molecular patterns of HAV in Tunisian population. The most recent study identified HAV strains circulating during 2008–2013 in North Central Tunisia and its migration pattern [31]. The present retrospective study aims to provide an overview of the genetic diversity of HAV strains circulating in Tunisia between 2013 and 2018, prior to the vaccine introduction into the national immunization program.

## 2. Materials and Methods

### 2.1. Samples

Out of 103 HAV IgM positive serum samples, 77 (74.8%) were included in this study. The inclusion criteria were positivity of both anti-HAV IgM and RNA detection by PCR amplification in the 5′UTR genomic regions (position: 55–678, according to reference strains HM175). The samples were collected as part of diagnostic activity of the Laboratory of Clinical Virology of Pasteur Institute of Tunis, between 2013 and 2018, and are originated from 7 districts; in the Northern, the Eastern and the central regions of the country. The samples were stored at −20 °C. No identifying patient data were used in this study.

### 2.2. RNA Extraction and RT-PCR

Viral RNA was extracted from 140 μL of serum using the QIAmp viral RNA mini Kit (QIAGEN, Hilden-Germany) following the manufacturer’s instructions. The RNA extracted was used as a template for the amplification of the complete VP1 gene (954 bp) and the VP1-2A junction (394bp) by nested RT-PCR. Reverse transcription and amplification were performed using previously published primers [8,31,32]. For both regions, the cDNA was synthetized using 10 µL of RNA and anti-sens primer m2: 5′-AGTCACACCTCTCCAGGAA-3′ and HAV3381: 5′-CCATYTCAAGAGTCCACACACT-3′ for the complete VP1 gene and the VP1-2A junction, respectively. The amplification of the VP1-2A junction was then carried out with 10 µL of cDNA and 0.2 µM of outer primers: HAV2870 (forward: 5′-GACAGATTCYACATTTGGATTGGT-3′) and HAV3381 (reverse: 5′-CCATYTCAAGAGTCCACACACT-3′) at 94 °C for 5 min followed by 35 cycles (30 s at 94 °C, 30 s at 58 °C, 1 min at 72 °C) and a final elongation at 72 °C for 5 min. Nested PCR was carried out with 5 µL of the product of the first PCR and 0.2 µM of inner primers HAV2896 (forward: 5′-CTATTCAGATTGCAAATTAYAAT-3′) and HAV3289 (reverse: 5′-AAYTTCATYATTTCATGCTCCT-3′), following the same thermal condition with the exception of the hybridation temperature 55 °C for the nested PCR. Total VP1 gene was amplified using outer primers HAV-2167 (forward: 5′-GTTTTGCTCCTCTTTATCATG-3′) and m2 (reverse: 5′-AGTCACACCTCTCCAGGAA-3′) and inner primers HAV m1 (forward: 5′-GCTCCTCTTTATCATGCTATG-3′) and HAV3125 (reverse: 5′-CCTGCATTCTATATGACTCT-3′), with same amplification duration for both PCR rounds: 94 °C for 5 min followed by 40 cycles (30 s at 94 °C, 30 s at 53 °C for first PCR or at 55 °C for the second PCR, 1 min at 72 °C) and a final elongation at 72 °C for 5 min. The PCR products were visualized on a 1% agarose gel.

### 2.3. Amplicon Purification and Sequencing

The PCR products of the VP1-2A junction and the whole VP1 were purified using the QIAquick PCR purification Kit (QIAGEN, GmnH, Hilden, Germany) following manufacturer’s instructions. Purified PCR-products were sequenced by inner primers of the nested PCR on an ABI Prism 3130-Genetic Analyser (3130-Genetic Analyser Applied Biosystems) using the Big Dye terminator ready reaction cycle sequencing Kit (Applied Biosystems).

### 2.4. Sequence Analyses and Genotyping

The sequence of each isolate was deduced by aligning the respective forward and reverse sequences using Chromas software version 2.6.2. Genotyping was performed using phylogenetic analysis comparing the VP1-2A and VP1 sequences with reference sequences representing the different HAV genotypes and sub-genotypes IA to IIIB. GenBank accessions numbers of references sequences used in this study were as follows: X75215, AF357222, AB020566 for genotype IA, M14707, M20273, AF314208 for genotype IB, HQ401240 for genotype IC, AY644676 for genotype IIA, AY644670 for genotype IIB, AB279733, AY644337 for genotype IIIA and AB279735, AB258387 for genotype IIIB. Different strains previously submitted in the nucleotide GenBank collection from Tunisia and other countries were selected, to study nucleotide and antigenic variability. Phylogenetic trees were constructed using MEGA software version 7.0.26 by the maximum likelihood method and the kimura-2 parameter model [33]. Topology was supported by 1000 bootstrap replicates. The new HAV sequences generated as part of the present work were submitted to GenBank database under accession numbers: MW117967 to MW118018 and MW222090 to MW222114 for the complete VP1 with a partial 2A region, and VP1-2A junction, respectively.

### 2.5. Molecular Modeling

The three-dimensional structure of HAV-VP1 protein (accession number: MW118004) was predicted using Modeller version 9.24 [34]. Its amino acid sequence was compared with other sequences retrieved from nrNCBI database using FASTA and BLAST. The Crystal structure of HAV (PDB code 4QPI) was used as a template, in which the VP1 protein (Chain_A) was extracted using MMTSB tool set [12]. The model corresponding to the best value of the DOPE score was selected after generating 1000 conformers [35]. All structures were visually explored using PyMOL molecular Viewer [36].

## 3. Results

### 3.1. Genetic and Phylogenetic Analysis

**Figure 1:**
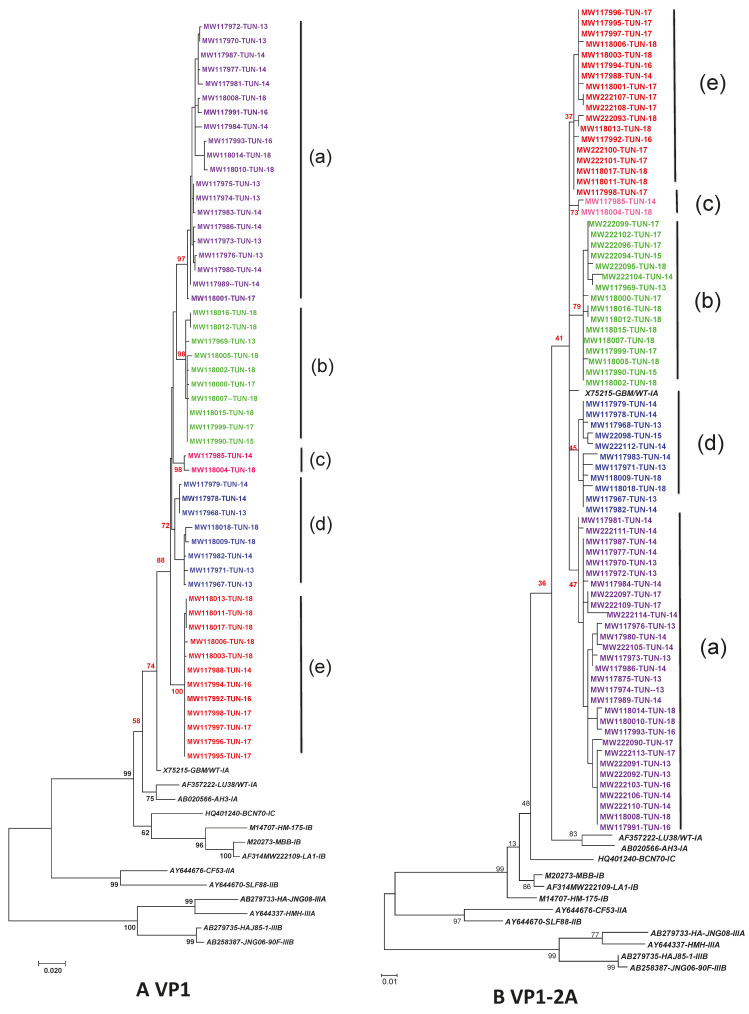
Phylogenetic trees comparing nucleotide sequences of HAV strains isolated in Tunisia during 2013–2018 with the reference sequences. The tree was performed using the maximum likelihood method and the kimura-2 parameter model. Topology was supported by 1000 bootstrap replicates. The sequences reported in this study were identified by the accession numbers. The sequences of isolates reported in this study are indicated by their accession number followed by the country code and the year of isolation. Reference HAV strains are shown in italic. Clusters are designed (**a**–**e**). The same color is assigned to the same sequences grouped in the same cluster. (**A**). Phylogenetic tree comparing 884nt of the VP1 region of 52 strains from this study with 12 reference HAV strains. (**B**). Phylogenetic tree comparing 319 nt of the VP1-2A region of 77 strains from this study with 12 reference HAV strains.

The 393 nucleotides in the VP1-2A junction could be amplified and sequenced from the 77 samples included in the study. Fifty-two complete sequences of the VP1 gene (954 nt long) were obtained. The phylogenetic trees in Figure 1 compare the Tunisian sequences in the entire VP1 region (Figure 1A) and the VP1-2A junction (Figure 1B) with reference sequences representative of the different HAV genotypes and subtypes. Both trees show that all isolates belonged to HAV genotype IA with similar grouping of the Tunisian sequences into 5 clusters (designed (a) to (d)); more consistent bootstrap values in the complete VP1 region were observed.

**Figure 2:**
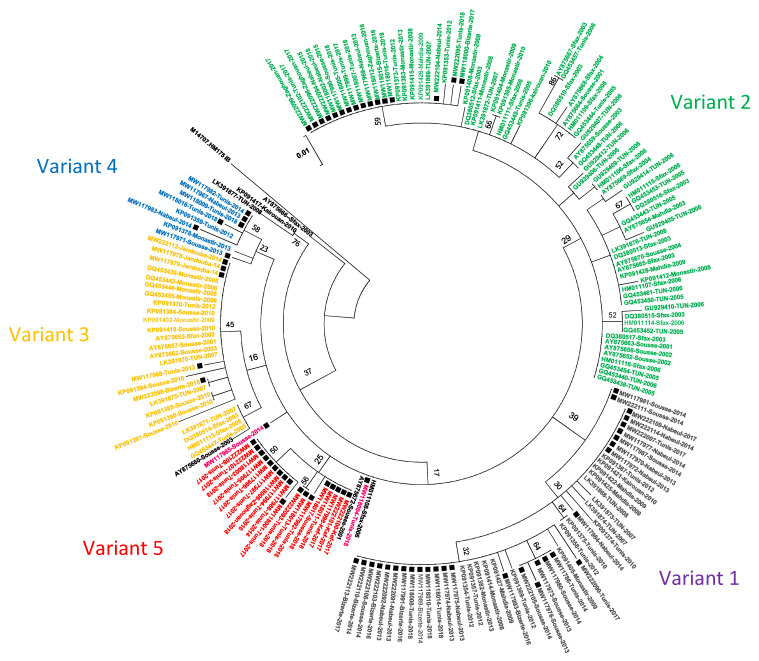
Phylogenetic tree of Tunisian HAV genotype IA isolates, constructed using the maximum likelihood method and the kimura-2 parameter model based on 168nt of the VP1-2A junction. Black squares represent isolates reported in this study (accession numbers MW117967 to MW118018 and MW222090 to MW222114). The sequences of isolates are indicated by their accession number followed by district and year of isolation. Sequences of unknown districts are indicated by their accession number followed by the country code and the year of isolation. Genetic variants are represented in different colors. Topology was supported by 1000 bootstrap replicates. Reference strain HM175 was used as an “outgroup”.

To better understand the molecular epidemiology of circulating strains in Tunisia, the 77 VP1-2A junctions detected in this work (2013–2018) were aligned with 104 previously published Tunisian strains of genotype IA collected during 2001–2013 period (Figure 2). The 104 sequences were selected among a total of 187 sequences published in Genbank. For identical sequences detected in the same year, only one representative sequence was selected for the phylogenetic analysis. Similar to Figure 1, the tree in Figure 2 shows the presence of at least five different variants that circulated in Tunisia between 2001 and 2018. A major variant (Variant 2) comprised strains that circulated in different regions of the country from 2001 to 2018. Variants 1, 3, 4 and 5 included sequences that were detected during somewhat shorter periods in 2007–2018, 2001–2015, 2012–2018 and 2014–2018, respectively.

**Figure 3:**
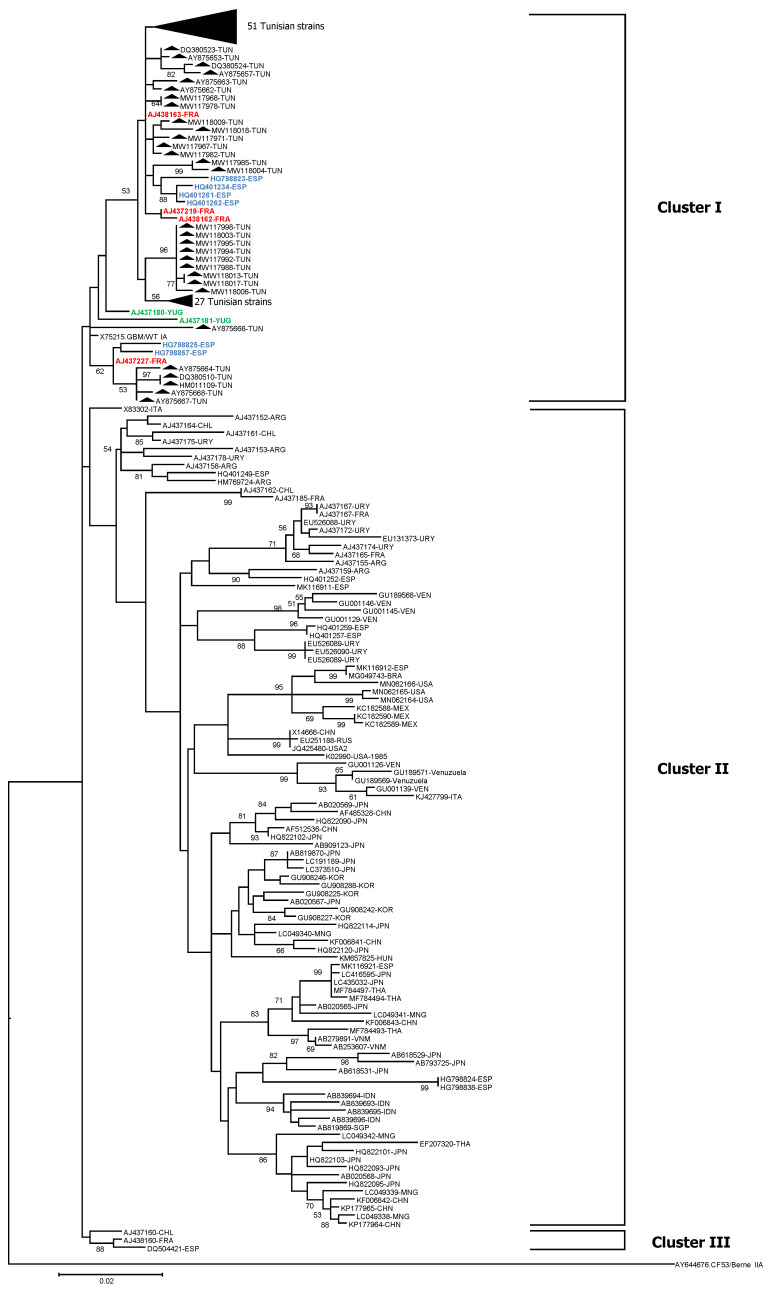
Phylogenetic tree based on the analysis of the whole VP1 region belonging to genotype IA, constructed using the maximum likelihood method and the kimura-2 parameter model. The tree includes HAV Tunisian 45 sequences reported in this study and 57 previously published sequences from Tunisia and 118 from other countries. Sequences from 21 different countries (Italy, France, Argentina, Chile, Uruguay, Spain, Venezuela, Brazil, USA, Mexico, China, Russia, Japan, South Korea, Mongolia, Hungary, Thailand, Vietnam, Indonesia, Singapore and Yugoslavia) were retrieved from GenBank. The sequences of isolates are indicated by their accession number and the country code. Black triangles represent Tunisian isolates. Sequences from others countries grouped with the Tunisian isolates are represented in different colors (sequences from France in red, from Spain in blue and from Yugoslavia in green). Topology was supported by 1000 bootstrap replicates. Bootstrap values lower than 50 were not indicated. Reference strain CF53 was used as an “outgroup”.

The phylogenetic comparison of the entire VP1 of HAV Tunisian strains with others published from 21 different countries of the world is shown in Figure 3. Based on the entire HAV VP1 sequence, the phylogenetic analysis shows the presence of three main clusters I to III. All the Tunisian sequences were grouped in one cluster (Cluster I) with sequences from France (4 sequences, represented in red), Spain (6 sequences, represented in blue) and Yugoslavia (2 sequences, represented in green). Cluster II enclosed sequences almost from all the different countries whereas the cluster III grouped only one sequence from Chile, one from France and one from Spain (Figure 3).

### 3.2. Amino Acid Analysis of the VP1 Protein

**Figure 4:**
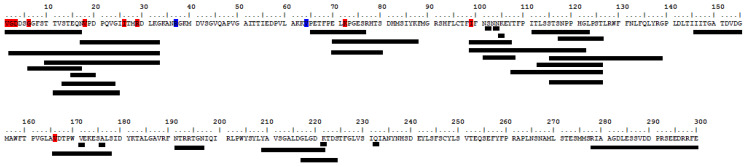
Amino acid replacements localization compared to reference strain and position within the experimentally B/Tcell epitopes regions [12,37,38,40,41,45]. Replacements located in epitope region are indicated in red. Replacements located close to epitope region are indicated in blue.

Amino acid analysis was performed for the 52 entire VP1 sequences obtained in this work by comparing the deduced amino acid with the prototype strain of genotype IA GBM/WT (accession numbers: X75215). Twelve different amino acid replacements were detected in the complete VP1 region (Table 1, Figure 4). Five were non-conservative replacements (position: VP1-1, VP1-2, VP1-6, VP1-26, VP1-99), one was semi-conservative (VP1-166) and the remaining ones were conservative (Table 1). The number of detected replacements varied from 3 to 5 per strain. Replacements VP1-26, VP1-37 and VP1-99 were found in all isolates; the one at position VP1-26 was reported as specific of genotype IB [37]. Figure 4 shows the location of replacements in relation to previously described epitope regions [38,39,40,41]. All amino acid replacements in the VP1 protein were located in or close to epitope regions (Figure 4). Ten of the amino acid replacements were located in B/T cell epitope regions 1–17, 6–17, 2–33, 10–33, 17–33, 11–25, 13–24, 15–20, 70–81, 99–107, 99–122 and 166–178 [12,37,38,39,40]. Replacement VP1-166 was located in epitope region 166–178 and also around the immunodominant site. Two replacements located in positions 37 and 64 were close to B/T cell epitope regions 10–33, 17–33 and 70–81. Among the detected amino acid replacements, only 2 out of the 12 replacements (positions 29 and 37) were previously described in Tunisia [42,43,44], the remaining ones are reported for the first time in the present work.

**Figure 5:**
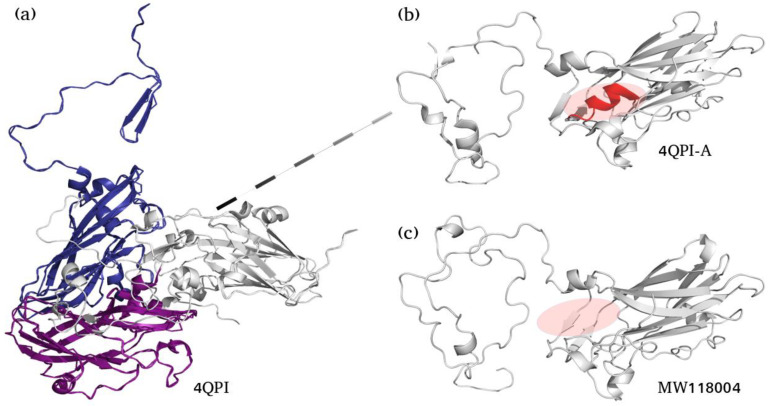
Effect of the deleted region found in the Tunisian variant on the three-dimensional structure of HAV-VP1 protein. (**a**) HAV capsid protein (PDB-ID: 4QPI). Gray protein corresponds to VP1, blue to VP2 and purple to VP3. (**b**) Top view Figure 1 protein (PDB-ID: 4QPI-A). (**c**) The three-dimensional predicted structure of the VP1 protein of the Tunisian variant isolated in 2018 (accession number: MW118004). The homology model was obtained using modeler and as a template HAV-VP1 protein (PDB: 4QPI-A). Circled region indicates the destroyed helix (colored in red) resulted from the deletion of 6 amino acids in the VP1 protein region.

In addition to the twelve amino acid replacements, a deletion of six amino acids RWFFNL (position 128 to 133) was found in one sequence (accession number: MW118004) located at the B/T cell epitope region 115–139. To study the effect of this deletion on the VP1 protein, a model of MW118004 was generated and selected according to several evaluation criteria. Figure 5 shows the three-dimensional structure of the VP1 of this isolate (Figure 5b) as compared to the template HAV-VP1 protein (PDB: 4QPI) (Figure 5a). The analysis of the three-dimensional structure, determined by homology modeling, shows that the deleted region found in the sequence of this isolate (PSTLRWFFNLF/PASTL- - - - - -F) resulted in a change in the VP1 protein structure by causing a destruction of an α-helix in the mature protein (Figure 5). This destroyed α-helix being located in an epitope region is likely to induce physicochemical modifications of the properties of this epitope, which may affect the binding affinity of the responding B cell and also of T cell, given that this deletion are located in a peptide recognized by T cell [38].

## 4. Discussion

This study reports an in-depth analysis of the genetic variability of HAV in Tunisia during the last few years. The sampling is not representative of hepatitis A cases in Tunisia due to the high frequency of asymptomatic forms and of symptomatic forms that are not reported. However, this study provides an overview on the molecular characterization of circulating strains. Phylogenetic analysis of HAV strains collected from different Tunisian districts during a six year period was conducted. All of the detected sequences belonged to genotype IA. The predominance of this genotype with the co-circulation of genotype IB was previously reported in Tunisia in both environmental and clinical samples [31,42,43,44,46,47]. The predominance of genotype IA was also described globally [48]. Same genotyping results were obtained based on the VP1-2A junction or the entire VP1 gene by phylogenetic analysis. Several studies showed that HAV genotyping could be performed using either the entire VP1 region, the N terminus of the VP1 region, the VP1–2A junction or the VP1–2B region [11,49,50,51,52]. Our results confirm that both regions can be used for HAV genotyping, although the reliability of bootstrap values in the phylogenetic analysis is more consistent when the entire VP1 sequences is used. Thus, VP1-2A junction remains a good solution for HAV genotyping, but for more in-depth analysis, larger genomic regions are more helpful.

To better understand the evolution and the genetic variability of circulating HAV sequences, we compared the VP1-2A junction sequences obtained in this work with others described in previous studies. Phylogenetic analyses showed that the Tunisian HAV strains detected between 2001 and 2018 were divided into at least five variants. A major variant (Variant2) included strains that circulated from 2001 to 2018 which seems have a continuous circulation in the country. The other variants were detected during shorter periods, 2007–2018 (Variant1), 2012–2018 (Variant4), 2014–2018 (Variant5) and 2001–2015 (Variant3). Variant3 was not detected after 2015. These findings confirm the co-circulation of different HAV variants within the genotype IA in multiple areas of Tunisia, which indicates that the endemicity level remains quite important. In fact, the number of genetic variants of pathogens generally decreases together with the progress of control strategies. While variant2 has continuous circulation, variant1, 3 and 5 were detected during shorter period, but it cannot be excluded that they may circulated during longer period given the lack of performant molecular surveillance during the study period.

When genotype IA Tunisian sequences, including the ones reported herein and previously published ones, were compared to HAV sequences from other countries, we found that all sequences from Tunisia grouped together in one cluster with other sequences from France, Spain and Yugoslavia. The second Cluster II enclosed strains from Asia, America and Europe whereas the cluster III grouped only sequences from Chile, France and Spain. These results suggest the presence of at least three IA variants within the IA subgenotype. However, the relatively limited number of sequences covering the total VP1 region available in GenBank, which originates from only 21 countries, may be the reason of the detection of only these three IA variants. Most of the published sequences cover a short size segment (<900 bases) of the genome which is not enough to properly determine the genetic variability of circulating strains. The availability in the future of full VP1 sequences from other countries in the world will help to better define the number of variants circulating worldwide and assess the genetic variability within genotype IA.

In the second part of the present work, we studied the impact of nucleotide mutations in the capsid protein VP1 gene on the amino-acid sequences to better understand the antigenic diversity of circulating HAV strains. The VP1 protein is a structural protein, known to contain major immunodominant epitopes of HAV [53,54]. VP1 protein analysis is important for predicting the possible antigenic escaping mutants within the circulating strains. Comparison of the deduced VP1 amino acid sequences obtained in the present study with the reference sequence of genotype IA showed several amino acid differences, mostly located in the N terminus of the VP1 region. Three to five different amino-acid mutations were identified per strain, all these mutations were located in or close to B/T cell epitope regions [12,37,38,40,41,45]. Most of the amino acid replacements were non-conservative and located in epitope regions. Thus, strains bearing such localized amino acid changes might be considered as potential antigenic variants. Eight amino acid replacements located in positions VP1-1, VP1-2, VP1-3, VP1-6, VP1-18, VP1-64, VP1-72 and VP1-99 were described for the first time in our study. Replacements VP1-26, VP1-37 and VP1-99 were found in all isolates of the present study. Replacement VP1-26 was identified previously in Argentina [49]. Replacement VP1-37 (R → K) was previously described in Tunisia and also in Argentina [36,37,38,49]. Replacement VP1-99 (I → T) is, to our knowledge reported for the first time in this study. This replacement same as replacements VP1-26 (R → K) and VP1-29 (R → K) seem to be associated to subtype IB [37]. In fact, replacement VP1-29 (R → K) was previously identified in Tunisia and in India and was associated widely to subtype IB [37]. Furthermore, recombination between sub-genotype IA and IB was previously described in Tunisia and other countries, which may explain this result [43,55].

A non-conservative replacement at position VP1-166 was previously identified in three isolates in Spain, during two outbreaks among male patients having sex with men; one was in non-vaccinated patient and the two others were in partly vaccinated patients [41,56]. These three isolates were considered as antigenic variants. In the present work, a semi-conservative amino acid replacement (V → A) at the same position was identified in one isolate detected in non-vaccinated patient, bearing four other amino acid replacements; two non-conservatives (in B/T cell epitope regions) and two conservatives (one in and the other close to B/T cell epitope regions). Thus, this isolate can be also considered as an antigenic variant.

These findings emphasize the importance of following the genetic variability of circulating strains to determinate the effects of vaccination on the molecular epidemiology of hepatitis A and on the potential selection of vaccine-escape mutants. Indeed, the emergence of potential antigenic variants, as well as vaccine-escape mutants after vaccine implementation, were previously described in many studies, especially when a man has sex with another man, and in immunocompromised patients [41,45,56,57,58,59]. In addition, it was previously reported that after vaccination amino acid replacement occurring in and around the epitopes regions was observed in vaccinated and unvaccinated persons [56]. The abundance of these amino-acid replacements was significantly higher in vaccinated patients, suggesting a positive selection of antigenic variants in some vaccinated patients [56]. Further molecular studies on HAV strains targeting the entire capsid proteins (VP1, VP2 and VP3) will be of great interest to better understand the evolution of circulating strains before and after introduction of HAV vaccine.

Interestingly, a deletion of 6 amino acids (PSTL- - - - - - F) position 128 to 133 was found in another isolate of the present study (accession N°: MW118004), located in the B/T cell epitope region (position 115–139) and resulted in the destruction of an α-helix (Figure 5) in the mature protein. Despite this deletion, the reading frame was conserved providing a non-defective virus as it was characterized by the presence of anti-HAV IgM antibody in symptomatic patient sera. Deletion mutants of VP1 were identified in other studies [44,45]. Aragones et al., 2007, studied HAV isolates evolution under a selective pressure of monoclonal antibodies and showed the occurrence of several deletion mutants, among which four contains the total or a part of the deleted region identified in the present study [45]. In Tunisia, Khelifi et al., reported another deletion in one isolate, a 38 amino acid deletion in VP1 protein (position 150–187) located in the part of the immunodominant antigenic site [44]. The occurrence of deletions seems to play a role in the adaptation to different selective pressures such as host defense mechanisms or new environmental conditions.

Despite the high degree of conservation of the capsid amino acid sequence shown by many studies, we found some degree of heterogeneity which revealed the circulation of several antigenic variants in the Tunisian HAV viral population. A previous study reported the circulation of two antigenic variants in Tunisia during 2003 [44]. One presented 38 amino acid deletions in the part of the immunodominant antigenic site of VP1 region and the second variant presented a replacement at the positionVP1-10. Our results suggest that a variety of mutants and antigenic variants circulates in Tunisia, despite the relatively low mutation rate reported for HAV, which seems to be related to the strict structural constraints of the viral capsid and a restricted codon usage [45,60].

## 5. Conclusions

The present study provides recent data on the molecular characteristics of circulating HAV strains in Tunisia. Genotype IA predominates, and several genetic and antigenic variants within this genotype co-circulate. Our study provides a baseline on the genetic variability of circulating strains before the introduction of the anti-HAV vaccines into the national immunization schedule. Strengthening the national hepatitis A infection surveillance system will be of great interest to monitor the impact of the vaccination program into HAV molecular epidemiology and the possible emergence of vaccine-escaping variants under selective pressures of this vaccination.

## Figures and Tables

**Table 1:** Amino acid replacements identified in Tunisian strains isolated during 2013–2018 and their locations.

Amino Acid Position	Prototype Residue	Substituted Residue	Type of Amino Acid Replacement	No. of Isolates
VP1-1	Val	Gly	Nonconservative	2
VP1-2	Gly	Glu	Nonconservative	1
VP1-3	Asp	Asn	Conservative	1
VP1-6	Gly	Glu	Nonconservative	1
VP1-18	Val	Ile	Conservative	1
VP1-26	Ile	Thr	Nonconservative	52
VP1-29	Arg	Lys	Conservative	6
VP1-37	Arg	Lys	Conservative	52
VP1-64	Val	Ile	Conservative	1
VP1-72	Lys	Arg	Conservative	1
VP1-99	Ile	Thr	Nonconservative	52
VP1-166	Val	Ala	Semiconservative	1

## Data Availability

The data that support the findings of this study are available from the corresponding author upon reasonable request.
